# Crystal structure of tetra­kis­(μ-caproato-κ^2^ *O*:*O*′)bis­[(4-cyano­pyridine-κ*N* ^1^)copper(II)]

**DOI:** 10.1107/S2056989015019052

**Published:** 2015-10-14

**Authors:** Sukanya Baruah, Zinnatara Islam, Sanjib Karmakar, Birinchi Kumar Das

**Affiliations:** a Department of Chemistry, Gauhati University, Guwahati 781 014, India; b Department of Instrumentation & USIC, Gauhati University, Guwahati 781 014, India

**Keywords:** crystal structure, dicopper complex, 4-cyano­pyridine, hexa­noic acid

## Abstract

The title dinuclear complex, [Cu_2_(C_6_H_11_O_2_)_4_(C_6_H_4_N_2_)_2_], has a paddle-wheel structure. The two crystallographically independent Cu^II^ atoms are each in a distorted square-pyramidal environment, in which four O atoms from the four bridging caproate ligands form the basal plane and the pyridine N atom of the 4-cyano­pyridine ligand occupies the apical position. The Cu⋯Cu distance is 2.6055 (9) Å. One of the alkyl chains of the caproate ligands is disordered over two sets of sites, with occupancies of 0.725 (5) and 0.275 (5). In the crystal, two pairs of C—H⋯N hydrogen bonds connect the mol­ecules into chains along [11-1] and C—H⋯O hydrogen bonds link the chains into a three-dimensional network.

## Related literature   

For related structures of copper(II) complexes, see: Brown & Chidambaram (1973 ▸); Petrič *et al.* (1995 ▸); Lomer & Perera (1974 ▸); Kozlevčar *et al.* (2000 ▸); Catterick & Thornton (1977 ▸). For applications as catalysts of dicopper(II) tetra­carboxyl­ates, see: Abied *et al.* (1987 ▸); Kozlevčar *et al.* (1999 ▸); Bora *et al.* (2007 ▸); Das *et al.* (2007 ▸); Sarmah *et al.* (2010 ▸).
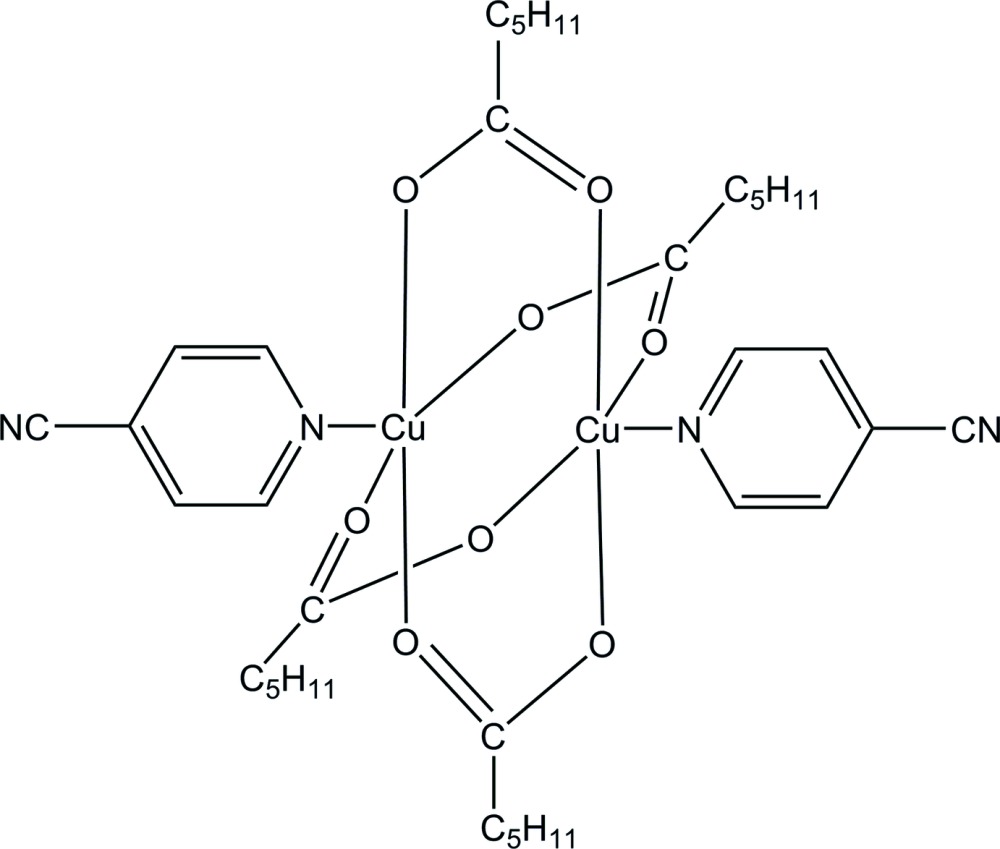


## Experimental   

### Crystal data  

[Cu_2_(C_6_H_11_O_2_)_4_(C_6_H_4_N_2_)_2_]
*M* *_r_* = 795.92
Monoclinic, 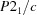

*a* = 8.7740 (4) Å
*b* = 25.3083 (11) Å
*c* = 17.7893 (8) Å
β = 101.321 (2)°
*V* = 3873.3 (3) Å^3^
*Z* = 4
Mo *K*α radiation
μ = 1.15 mm^−1^
*T* = 100 K
0.42 × 0.18 × 0.14 mm

### Data collection  

Bruker SMART APEXII CCD diffractometer
Absorption correction: multi-scan (*SADABS*; Sheldrick, 1996 ▸) *T*_min_ = 0.780, *T*_max_ = 0.851
29777 measured reflections
11382 independent reflections
8952 reflections with *I* > 2σ(*I*)
*R*_int_ = 0.032

### Refinement  

*R*[*F* ^2^ > 2σ(*F* ^2^)] = 0.035
*wR*(*F* ^2^) = 0.084
*S* = 1.03
11382 reflections
502 parameters
155 restraints
H-atom parameters constrained
Δρ_max_ = 0.47 e Å^−3^
Δρ_min_ = −0.40 e Å^−3^

### 

Data collection: *APEX2* (Bruker, 2004 ▸); cell refinement: *SAINT* (Bruker, 2004 ▸); data reduction: *SAINT*; program(s) used to solve structure: *SHELXS97* (Sheldrick, 2008 ▸); program(s) used to refine structure: *SHELXL2012/9* (Sheldrick, 2015 ▸); molecular graphics: *ORTEP-3 for Windows* (Farrugia, 2012 ▸) and *Mercury* (Macrae *et al.*, 2008 ▸); software used to prepare material for publication: *SHELXL2012/9*.

## Supplementary Material

Crystal structure: contains datablock(s) I, Global. DOI: 10.1107/S2056989015019052/is5420sup1.cif

Structure factors: contains datablock(s) I. DOI: 10.1107/S2056989015019052/is5420Isup2.hkl

**Fig. 1.**
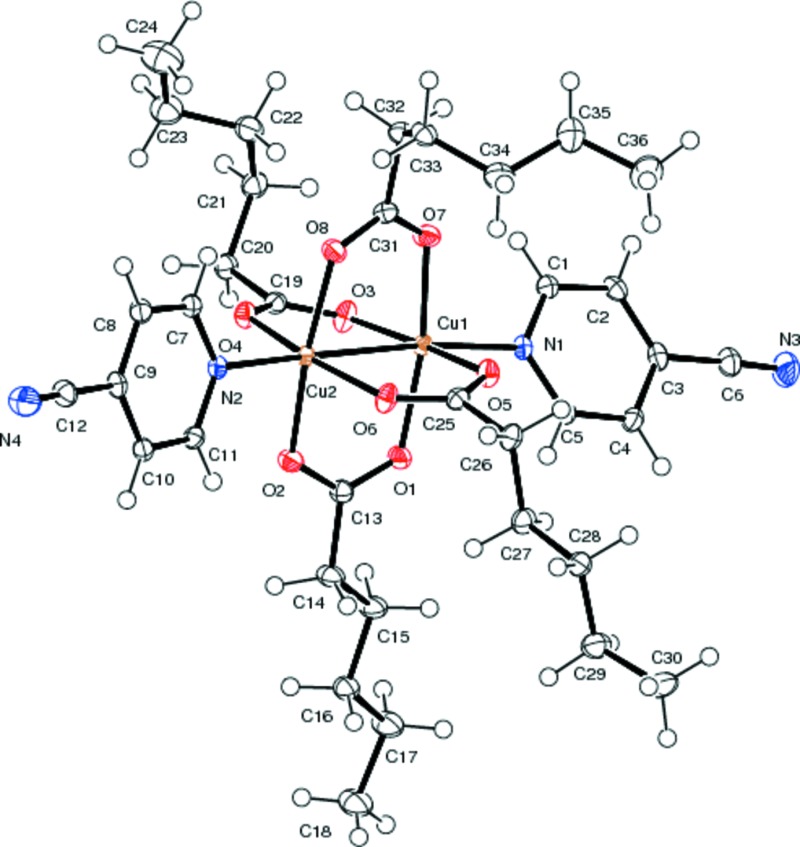
Molecular structure of the title compound drawn with 30% probability ellipsoid. H-atoms are shown as circles of arbitrary radius. Only one component of the disordered alkyl chain is shown.

. DOI: 10.1107/S2056989015019052/is5420fig1.tif
Mol­ecular structure of the title compound drawn with 30% probability ellipsoid. H-atoms are shown as circles of arbitrary radius. Only one component of the disordered alkyl chain is shown.

**Fig. 2.**
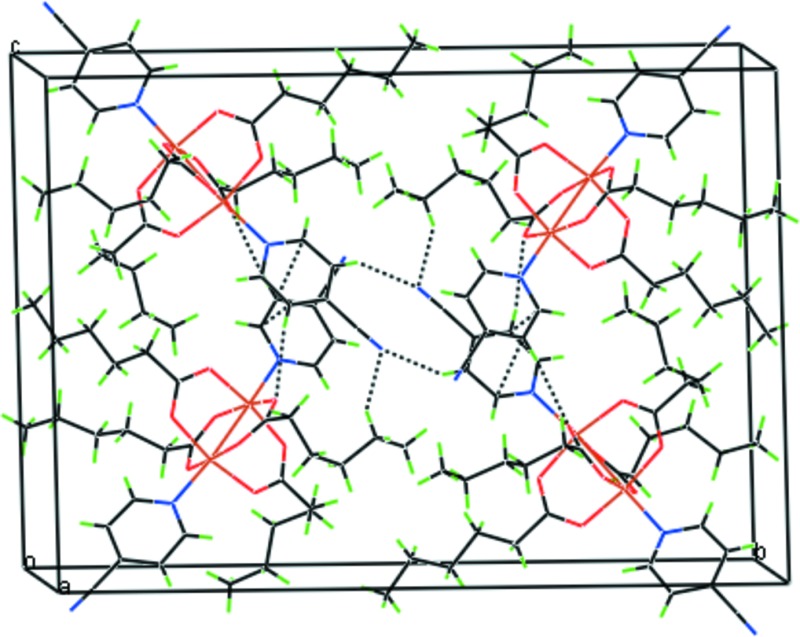
A packing diagram of the title compound. Hydrogen bonds are shown by dotted lines.

. DOI: 10.1107/S2056989015019052/is5420fig2.tif
A packing diagram of the title compound. Hydrogen bonds are shown by dotted lines.

CCDC reference: 1430487

Additional supporting information:  crystallographic information; 3D view; checkCIF report

## Figures and Tables

**Table 1:** Hydrogen-bond geometry (, )

*D*H*A*	*D*H	H*A*	*D* *A*	*D*H*A*
C2H2O4^i^	0.95	2.55	3.301(2)	137
C4H4N3^ii^	0.95	2.58	3.444(2)	151
C8H8N4^iii^	0.95	2.48	3.422(2)	169
C10H10O5^iv^	0.95	2.59	3.432(2)	148
C20H20*B*O6^v^	0.99	2.66	3.479(2)	141
C26H26*A*O3^vi^	0.99	2.56	3.532(2)	167

Symmetry codes: (i) 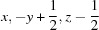; (ii) 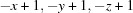; (iii) 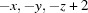; (iv) 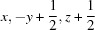; (v) 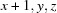; (vi) 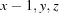.

## supplementary crystallographic information

### S1. Comment

A few members of the family of dicopper(II) tetracarboxylates of the type Cu_2_(µ-O_2_CR)_4_*L*_2_, where *R* is either an alkyl or aryl group and *L* is pyridine or a pyridyl ligand, have been demonstrated as homogeneous catalysts in the oxidation of various alcohols (Abied *et al.*, 1987; Kozlevčar *et al.*, 1999; Bora *et al.*, 2007; Das *et al.*, 2007; Sarmah *et al.*, 2010). In view of this it was found instructive to prepare other members of the above general formula having *R* = a long-chain alkyl group because the presence such alkyl groups could make the resultant dimeric carboxylates more soluble in organic solvents, and hence more effective as catalysts.

We aimed to prepare complexes of long chain carboxylic acids as only a few crystal structures of such complexes of copper(II) have been reported (Petrič *et al.*, 1995; Lomer & Perera, 1974). The structure of the title compound, [Cu_2_(µ-O_2_CC_5_H_11_)_4_(4-CNpy)_2_], (I), is similar to that of copper(II) acetate hydrate (Brown & Chidambaram, 1973). The Cu—Cu distance of 2.6055 (9) Å is shorter than the corresponding distance in [Cu_2_(µ-O_2_CCH_3_)_4_(H_2_O)_2_] (2.614 Å) as well as that in [Cu_2_(OOCC_5_H_11_)_4_(OCN_2_H_4_)_2_] [2.644 (2) Å] (Kozlevčar *et al.*, 2000). The average Cu—O bond length of 1.9731 (12) Å and the longer average Cu—N distance of 2.1837 (13) Å in the title complex are considered to be normal for [Cu_2_(µ-O_2_CR)_4_*L*_2_] (*L* = axial ligand, *R* = alkyl group) type of structures (Catterick & Thornton, 1977). In (I), one pair of hydrocarbon chains has the common zigzag conformation while the other pair is distorted, which facilitates efficient packing.

### S2. Experimental

CuSO_4_·5H_2_O (0.749 g, 3 mmol) was dissolved in methanol (25 ml). To this solution, sodium caproate (C_5_H_11_COONa; 0.882 g, 6 mmol) and 4-cyanopyridine (0.321 g, 3 mmol) were added and the mixture was stirred for 2 h. The resulting green product was filtered off, washed with small volumes of methanol and dried in a vacuum desiccator over fused CaCl_2_ (yield 80%). The product was dissolved in acetonitrile to get a greenish homogeneous solution which was allowed to concentrate by evaporation at room temperature. Single crystals suitable for X-ray diffraction were obtained from this solution after one day and collected by filtration.

### S3. Refinement

H atoms were located in a difference Fourier map and were subsequently treated as riding with C—H = 0.95–0.99 Å, and with *U*_iso_(H) = 1.2*U*_eq_(C) or 1.5*U*_eq_(methyl C). One of the alkyl chains of caproato ligands is disordered over two sites with refined occupancies of 0.725 (5) and 0.275 (5). Restraints of same displacement parameters (*SIMU*) and same distances (*SADI*) were applied for the disordered C atoms, C32–C36 and C32'–C36'.

### Crystal data

**Table:**

[Cu_2_(C_6_H_11_O_2_)_4_(C_6_H_4_N_2_)_2_]	*F*(000) = 1672
*M**_r_* = 795.92	*D*_x_ = 1.365 Mg m^−^^3^
Monoclinic, *P*2_1_/*c*	Mo *K*α radiation, λ = 0.71073 Å
*a* = 8.7740 (4) Å	Cell parameters from 6882 reflections
*b* = 25.3083 (11) Å	θ = 2.6–28.2°
*c* = 17.7893 (8) Å	µ = 1.15 mm^−^^1^
β = 101.321 (2)°	*T* = 100 K
*V* = 3873.3 (3) Å^3^	Plate, green
*Z* = 4	0.42 × 0.18 × 0.14 mm

### Data collection

**Table:**

Bruker SMART APEXII CCD diffractometer	8952 reflections with *I* > 2σ(*I*)
Radiation source: fine-focus sealed tube	*R*_int_ = 0.032
ω and φ scan	θ_max_ = 30.1°, θ_min_ = 2.3°
Absorption correction: multi-scan (*SADABS*; Sheldrick, 1996)	*h* = −12→12
*T*_min_ = 0.780, *T*_max_ = 0.851	*k* = −35→27
29777 measured reflections	*l* = −25→20
11382 independent reflections	

### Refinement

**Table:**

Refinement on *F*^2^	Primary atom site location: structure-invariant direct methods
Least-squares matrix: full	Secondary atom site location: difference Fourier map
*R*[*F*^2^ > 2σ(*F*^2^)] = 0.035	Hydrogen site location: inferred from neighbouring sites
*wR*(*F*^2^) = 0.084	H-atom parameters constrained
*S* = 1.03	*w* = 1/[σ^2^(*F*_o_^2^) + (0.0362*P*)^2^ + 1.043*P*] where *P* = (*F*_o_^2^ + 2*F*_c_^2^)/3
11382 reflections	(Δ/σ)_max_ = 0.002
502 parameters	Δρ_max_ = 0.47 e Å^−^^3^
155 restraints	Δρ_min_ = −0.40 e Å^−^^3^

### Special details

**Table:**

Geometry. All e.s.d.'s (except the e.s.d. in the dihedral angle between two l.s. planes) are estimated using the full covariance matrix. The cell e.s.d.'s are taken into account individually in the estimation of e.s.d.'s in distances, angles and torsion angles; correlations between e.s.d.'s in cell parameters are only used when they are defined by crystal symmetry. An approximate (isotropic) treatment of cell e.s.d.'s is used for estimating e.s.d.'s involving l.s. planes.
Refinement. One of the four pentane side chains was found to be two fold disordered·The components of disorder could be completed through successive difference fourier·The two components were refined with sum of their occupancies restrained as 1. Also the bond distances and thermal parameters of the disordered components were restrained to be with in chemically meaningful range. Finally when the refinement converged the relative occupancies were 0.725 and 0.275. The alkyl and aromatic H atoms were allowed to ride at a distance of 0.99 Å and 0.95 Å respectively during refinement.

### Fractional atomic coordinates and isotropic or equivalent isotropic displacement parameters (Å^2^)

**Table:**

	*x*	*y*	*z*	*U*_iso_*/*U*_eq_	Occ. (<1)
C1	0.60748 (19)	0.30675 (7)	0.58958 (9)	0.0163 (3)	
H1	0.6381	0.2711	0.5851	0.020*	
C2	0.6355 (2)	0.34294 (7)	0.53544 (10)	0.0177 (3)	
H2	0.6834	0.3324	0.4943	0.021*	
C3	0.59136 (19)	0.39498 (7)	0.54309 (9)	0.0164 (3)	
C4	0.52020 (19)	0.40919 (7)	0.60344 (9)	0.0166 (3)	
H4	0.4885	0.4446	0.6094	0.020*	
C5	0.49707 (19)	0.37039 (7)	0.65427 (9)	0.0155 (3)	
H5	0.4490	0.3798	0.6958	0.019*	
C6	0.6159 (2)	0.43498 (8)	0.48848 (10)	0.0223 (4)	
C7	0.24481 (19)	0.10600 (7)	0.89762 (9)	0.0157 (3)	
H7	0.2710	0.0899	0.8536	0.019*	
C8	0.16697 (19)	0.07593 (7)	0.94337 (9)	0.0172 (3)	
H8	0.1395	0.0402	0.9312	0.021*	
C9	0.13064 (19)	0.10037 (7)	1.00806 (9)	0.0157 (3)	
C10	0.17279 (19)	0.15263 (7)	1.02468 (9)	0.0152 (3)	
H10	0.1501	0.1696	1.0688	0.018*	
C11	0.24889 (18)	0.17907 (7)	0.97479 (9)	0.0143 (3)	
H11	0.2771	0.2150	0.9852	0.017*	
C12	0.0483 (2)	0.07087 (7)	1.05751 (10)	0.0202 (4)	
C13	0.48541 (19)	0.30546 (7)	0.88176 (10)	0.0165 (3)	
C14	0.5101 (2)	0.34881 (7)	0.94087 (10)	0.0217 (4)	
H14A	0.5642	0.3336	0.9902	0.026*	
H14B	0.4069	0.3612	0.9482	0.026*	
C15	0.6021 (2)	0.39649 (7)	0.92292 (10)	0.0223 (4)	
H15A	0.6977	0.3843	0.9065	0.027*	
H15B	0.5392	0.4165	0.8800	0.027*	
C16	0.6459 (2)	0.43256 (7)	0.99196 (10)	0.0220 (4)	
H16A	0.5497	0.4435	1.0090	0.026*	
H16B	0.7098	0.4122	1.0343	0.026*	
C17	0.7348 (3)	0.48173 (8)	0.97790 (11)	0.0295 (4)	
H17A	0.8284	0.4712	0.9582	0.035*	
H17B	0.6688	0.5035	0.9381	0.035*	
C18	0.7842 (3)	0.51469 (8)	1.04962 (11)	0.0332 (5)	
H18A	0.6923	0.5244	1.0703	0.050*	
H18B	0.8363	0.5468	1.0370	0.050*	
H18C	0.8558	0.4942	1.0880	0.050*	
C19	0.70285 (19)	0.19658 (7)	0.81562 (9)	0.0154 (3)	
C20	0.86267 (19)	0.17169 (7)	0.82889 (10)	0.0176 (3)	
H20A	0.8726	0.1458	0.8713	0.021*	
H20B	0.9427	0.1993	0.8439	0.021*	
C21	0.8892 (2)	0.14391 (8)	0.75616 (10)	0.0226 (4)	
H21A	0.8788	0.1702	0.7143	0.027*	
H21B	0.9969	0.1302	0.7653	0.027*	
C22	0.7773 (2)	0.09835 (8)	0.73012 (11)	0.0253 (4)	
H22A	0.6694	0.1113	0.7254	0.030*	
H22B	0.7903	0.0867	0.6786	0.030*	
C23	0.8004 (2)	0.05124 (8)	0.78378 (12)	0.0297 (4)	
H23A	0.7936	0.0633	0.8360	0.036*	
H23B	0.9061	0.0368	0.7860	0.036*	
C24	0.6826 (3)	0.00750 (10)	0.75984 (13)	0.0411 (6)	
H24A	0.5784	0.0204	0.7624	0.062*	
H24B	0.7083	−0.0227	0.7944	0.062*	
H24C	0.6849	−0.0034	0.7072	0.062*	
C25	0.15800 (19)	0.27344 (7)	0.74169 (9)	0.0150 (3)	
C26	0.00487 (19)	0.30213 (7)	0.72607 (10)	0.0174 (3)	
H26A	−0.0743	0.2813	0.7458	0.021*	
H26B	−0.0310	0.3069	0.6702	0.021*	
C27	0.0260 (2)	0.35598 (7)	0.76576 (10)	0.0198 (4)	
H27A	0.0592	0.3505	0.8217	0.024*	
H27B	0.1097	0.3755	0.7477	0.024*	
C28	−0.12088 (19)	0.38928 (7)	0.75090 (10)	0.0184 (3)	
H28A	−0.2024	0.3710	0.7725	0.022*	
H28B	−0.1586	0.3926	0.6949	0.022*	
C29	−0.0954 (2)	0.44412 (8)	0.78583 (11)	0.0252 (4)	
H29A	−0.0742	0.4409	0.8424	0.030*	
H29B	−0.0025	0.4600	0.7710	0.030*	
C30	−0.2329 (2)	0.48086 (8)	0.76122 (12)	0.0325 (5)	
H30A	−0.2500	0.4863	0.7056	0.049*	
H30B	−0.2114	0.5149	0.7874	0.049*	
H30C	−0.3260	0.4651	0.7747	0.049*	
C31	0.36022 (19)	0.16115 (7)	0.68197 (10)	0.0166 (3)	
C32	0.2997 (7)	0.1186 (4)	0.6233 (6)	0.0184 (13)	0.725 (5)
H32A	0.3523	0.0848	0.6405	0.022*	0.725 (5)
H32B	0.3279	0.1282	0.5739	0.022*	0.725 (5)
C33	0.1248 (4)	0.11020 (16)	0.6104 (2)	0.0188 (7)	0.725 (5)
H33A	0.0965	0.1005	0.6598	0.023*	0.725 (5)
H33B	0.0966	0.0802	0.5748	0.023*	0.725 (5)
C34	0.0300 (3)	0.15831 (11)	0.57799 (15)	0.0245 (7)	0.725 (5)
H34A	−0.0800	0.1524	0.5814	0.029*	0.725 (5)
H34B	0.0675	0.1894	0.6101	0.029*	0.725 (5)
C35	0.0382 (4)	0.17068 (13)	0.49539 (17)	0.0380 (9)	0.725 (5)
H35A	0.0119	0.1384	0.4642	0.046*	0.725 (5)
H35B	0.1462	0.1806	0.4929	0.046*	0.725 (5)
C36	−0.0707 (9)	0.2152 (2)	0.4604 (3)	0.0436 (13)	0.725 (5)
H36A	−0.1766	0.2074	0.4671	0.065*	0.725 (5)
H36B	−0.0695	0.2181	0.4055	0.065*	0.725 (5)
H36C	−0.0358	0.2486	0.4859	0.065*	0.725 (5)
C32'	0.3276 (19)	0.1158 (10)	0.6250 (17)	0.014 (3)	0.275 (5)
H32C	0.3859	0.1200	0.5830	0.016*	0.275 (5)
H32D	0.3537	0.0813	0.6504	0.016*	0.275 (5)
C33'	0.1558 (13)	0.1214 (4)	0.5961 (6)	0.026 (2)	0.275 (5)
H33C	0.1033	0.1199	0.6405	0.031*	0.275 (5)
H33D	0.1193	0.0907	0.5629	0.031*	0.275 (5)
C34'	0.1052 (8)	0.1717 (3)	0.5512 (4)	0.0249 (17)	0.275 (5)
H34C	0.1350	0.2023	0.5856	0.030*	0.275 (5)
H34D	0.1632	0.1744	0.5089	0.030*	0.275 (5)
C35'	−0.0658 (9)	0.1753 (3)	0.5179 (5)	0.040 (2)	0.275 (5)
H35C	−0.0963	0.1461	0.4810	0.049*	0.275 (5)
H35D	−0.1257	0.1717	0.5593	0.049*	0.275 (5)
C36'	−0.103 (2)	0.2273 (5)	0.4779 (9)	0.048 (4)	0.275 (5)
H36D	−0.0707	0.2562	0.5143	0.073*	0.275 (5)
H36E	−0.2153	0.2296	0.4581	0.073*	0.275 (5)
H36F	−0.0477	0.2300	0.4353	0.073*	0.275 (5)
N1	0.53919 (15)	0.31990 (6)	0.64800 (8)	0.0139 (3)	
N2	0.28465 (15)	0.15633 (5)	0.91233 (8)	0.0139 (3)	
N3	0.6346 (2)	0.46784 (7)	0.44711 (10)	0.0327 (4)	
N4	−0.0195 (2)	0.04841 (7)	1.09612 (9)	0.0286 (4)	
O1	0.49867 (14)	0.31614 (5)	0.81437 (7)	0.0187 (3)	
O2	0.45038 (14)	0.26065 (5)	0.90499 (7)	0.0188 (3)	
O3	0.67962 (13)	0.23458 (5)	0.76840 (7)	0.0201 (3)	
O4	0.60184 (13)	0.17736 (5)	0.84962 (7)	0.0174 (2)	
O5	0.25478 (13)	0.28500 (5)	0.69930 (7)	0.0179 (2)	
O6	0.18508 (13)	0.24217 (5)	0.79778 (7)	0.0189 (3)	
O7	0.42718 (15)	0.20116 (5)	0.66123 (7)	0.0204 (3)	
O8	0.32866 (14)	0.15394 (5)	0.74747 (7)	0.0183 (3)	
Cu1	0.47349 (2)	0.26351 (2)	0.72892 (2)	0.01245 (5)	
Cu2	0.38502 (2)	0.20385 (2)	0.83178 (2)	0.01225 (5)	

### Atomic displacement parameters (Å^2^)

**Table:**

	*U*^11^	*U*^22^	*U*^33^	*U*^12^	*U*^13^	*U*^23^
C1	0.0175 (8)	0.0161 (8)	0.0153 (8)	0.0002 (6)	0.0037 (6)	0.0009 (6)
C2	0.0192 (8)	0.0212 (9)	0.0132 (8)	−0.0029 (7)	0.0045 (7)	0.0000 (7)
C3	0.0146 (8)	0.0197 (9)	0.0137 (8)	−0.0047 (6)	−0.0004 (6)	0.0043 (7)
C4	0.0174 (8)	0.0132 (8)	0.0181 (8)	−0.0003 (6)	0.0006 (7)	0.0021 (6)
C5	0.0143 (8)	0.0169 (8)	0.0151 (8)	−0.0002 (6)	0.0024 (6)	0.0006 (6)
C6	0.0249 (9)	0.0231 (9)	0.0187 (9)	−0.0010 (7)	0.0040 (7)	0.0039 (7)
C7	0.0185 (8)	0.0143 (8)	0.0137 (8)	0.0000 (6)	0.0023 (6)	0.0012 (6)
C8	0.0191 (8)	0.0148 (8)	0.0162 (8)	−0.0038 (6)	−0.0001 (7)	0.0010 (6)
C9	0.0141 (8)	0.0186 (8)	0.0140 (8)	−0.0020 (6)	0.0019 (6)	0.0038 (6)
C10	0.0153 (8)	0.0172 (8)	0.0132 (7)	0.0017 (6)	0.0029 (6)	0.0019 (6)
C11	0.0141 (7)	0.0127 (8)	0.0158 (8)	−0.0009 (6)	0.0023 (6)	0.0010 (6)
C12	0.0227 (9)	0.0199 (9)	0.0182 (8)	−0.0042 (7)	0.0046 (7)	0.0013 (7)
C13	0.0169 (8)	0.0165 (8)	0.0164 (8)	−0.0008 (6)	0.0043 (7)	−0.0003 (6)
C14	0.0322 (10)	0.0165 (9)	0.0179 (8)	−0.0068 (7)	0.0087 (8)	−0.0037 (7)
C15	0.0325 (10)	0.0156 (8)	0.0204 (9)	−0.0055 (7)	0.0090 (8)	−0.0035 (7)
C16	0.0308 (10)	0.0172 (9)	0.0183 (9)	−0.0037 (7)	0.0055 (8)	−0.0011 (7)
C17	0.0466 (12)	0.0206 (10)	0.0233 (10)	−0.0119 (9)	0.0113 (9)	−0.0036 (8)
C18	0.0511 (13)	0.0228 (10)	0.0262 (10)	−0.0143 (9)	0.0086 (10)	−0.0058 (8)
C19	0.0150 (8)	0.0144 (8)	0.0158 (8)	−0.0002 (6)	0.0007 (6)	−0.0014 (6)
C20	0.0140 (8)	0.0195 (9)	0.0184 (8)	0.0017 (6)	0.0007 (7)	0.0008 (7)
C21	0.0223 (9)	0.0238 (9)	0.0226 (9)	0.0058 (7)	0.0064 (7)	0.0019 (8)
C22	0.0250 (10)	0.0291 (10)	0.0209 (9)	0.0052 (8)	0.0023 (8)	−0.0044 (8)
C23	0.0316 (11)	0.0275 (11)	0.0303 (11)	−0.0058 (9)	0.0067 (9)	−0.0024 (9)
C24	0.0443 (13)	0.0426 (14)	0.0389 (13)	−0.0172 (11)	0.0144 (11)	−0.0106 (11)
C25	0.0139 (8)	0.0133 (8)	0.0176 (8)	−0.0014 (6)	0.0026 (6)	−0.0003 (6)
C26	0.0135 (8)	0.0180 (8)	0.0208 (8)	0.0018 (6)	0.0033 (7)	0.0031 (7)
C27	0.0164 (8)	0.0202 (9)	0.0217 (9)	0.0028 (7)	0.0012 (7)	0.0003 (7)
C28	0.0164 (8)	0.0200 (9)	0.0183 (8)	0.0030 (7)	0.0024 (7)	0.0000 (7)
C29	0.0246 (9)	0.0238 (10)	0.0269 (10)	0.0030 (8)	0.0040 (8)	−0.0046 (8)
C30	0.0368 (12)	0.0248 (10)	0.0359 (11)	0.0094 (9)	0.0077 (9)	−0.0070 (9)
C31	0.0176 (8)	0.0152 (8)	0.0176 (8)	0.0013 (6)	0.0048 (7)	−0.0020 (7)
C32	0.021 (3)	0.018 (2)	0.0169 (19)	0.002 (2)	0.007 (2)	−0.0019 (15)
C33	0.0200 (16)	0.0190 (17)	0.0170 (16)	−0.0056 (12)	0.0023 (12)	−0.0026 (12)
C34	0.0205 (13)	0.0258 (14)	0.0264 (14)	−0.0032 (11)	0.0025 (11)	−0.0049 (11)
C35	0.052 (2)	0.0336 (17)	0.0294 (17)	0.0076 (15)	0.0090 (15)	0.0086 (13)
C36	0.060 (3)	0.027 (3)	0.040 (3)	−0.001 (2)	0.001 (2)	0.003 (2)
C32'	0.012 (5)	0.012 (5)	0.020 (5)	0.002 (4)	0.009 (4)	−0.005 (4)
C33'	0.037 (5)	0.020 (4)	0.024 (4)	−0.015 (3)	0.013 (4)	−0.010 (3)
C34'	0.027 (4)	0.024 (3)	0.021 (3)	−0.004 (3)	−0.004 (3)	−0.002 (3)
C35'	0.032 (4)	0.038 (4)	0.042 (5)	−0.010 (4)	−0.015 (4)	0.004 (4)
C36'	0.066 (10)	0.031 (7)	0.037 (7)	−0.003 (6)	−0.017 (6)	0.008 (6)
N1	0.0129 (6)	0.0150 (7)	0.0133 (6)	−0.0012 (5)	0.0011 (5)	0.0029 (5)
N2	0.0129 (6)	0.0142 (7)	0.0139 (6)	0.0001 (5)	0.0011 (5)	0.0024 (5)
N3	0.0436 (11)	0.0296 (10)	0.0278 (9)	0.0016 (8)	0.0139 (8)	0.0109 (8)
N4	0.0367 (9)	0.0242 (9)	0.0283 (9)	−0.0082 (7)	0.0144 (8)	−0.0001 (7)
O1	0.0262 (7)	0.0158 (6)	0.0146 (6)	−0.0024 (5)	0.0053 (5)	0.0003 (5)
O2	0.0260 (6)	0.0155 (6)	0.0160 (6)	−0.0053 (5)	0.0066 (5)	−0.0012 (5)
O3	0.0140 (6)	0.0200 (6)	0.0270 (7)	0.0019 (5)	0.0055 (5)	0.0093 (5)
O4	0.0161 (6)	0.0194 (6)	0.0170 (6)	0.0019 (5)	0.0044 (5)	0.0040 (5)
O5	0.0145 (6)	0.0217 (6)	0.0184 (6)	0.0020 (5)	0.0050 (5)	0.0052 (5)
O6	0.0156 (6)	0.0184 (6)	0.0238 (6)	0.0021 (5)	0.0066 (5)	0.0076 (5)
O7	0.0284 (7)	0.0164 (6)	0.0188 (6)	−0.0026 (5)	0.0103 (5)	−0.0018 (5)
O8	0.0236 (6)	0.0170 (6)	0.0144 (6)	−0.0035 (5)	0.0039 (5)	−0.0006 (5)
Cu1	0.01285 (10)	0.01172 (10)	0.01342 (10)	0.00012 (7)	0.00414 (7)	0.00229 (7)
Cu2	0.01339 (10)	0.01170 (10)	0.01211 (10)	−0.00084 (7)	0.00359 (7)	0.00147 (7)

### Geometric parameters (Å, º)

**Table:**

C1—N1	1.340 (2)	C25—O5	1.2754 (18)
C1—C2	1.385 (2)	C25—C26	1.504 (2)
C1—H1	0.9500	C26—C27	1.529 (2)
C2—C3	1.387 (2)	C26—H26A	0.9900
C2—H2	0.9500	C26—H26B	0.9900
C3—C4	1.391 (2)	C27—C28	1.519 (2)
C3—C6	1.448 (2)	C27—H27A	0.9900
C4—C5	1.377 (2)	C27—H27B	0.9900
C4—H4	0.9500	C28—C29	1.519 (3)
C5—N1	1.341 (2)	C28—H28A	0.9900
C5—H5	0.9500	C28—H28B	0.9900
C6—N3	1.143 (2)	C29—C30	1.518 (3)
C7—N2	1.333 (2)	C29—H29A	0.9900
C7—C8	1.388 (2)	C29—H29B	0.9900
C7—H7	0.9500	C30—H30A	0.9800
C8—C9	1.398 (2)	C30—H30B	0.9800
C8—H8	0.9500	C30—H30C	0.9800
C9—C10	1.389 (2)	C31—O7	1.262 (2)
C9—C12	1.450 (2)	C31—O8	1.2627 (19)
C10—C11	1.383 (2)	C31—C32'	1.520 (14)
C10—H10	0.9500	C31—C32	1.520 (6)
C11—N2	1.342 (2)	C32—C33	1.521 (7)
C11—H11	0.9500	C32—H32A	0.9900
C12—N4	1.144 (2)	C32—H32B	0.9900
C13—O1	1.2564 (19)	C33—C34	1.523 (5)
C13—O2	1.265 (2)	C33—H33A	0.9900
C13—C14	1.506 (2)	C33—H33B	0.9900
C14—C15	1.520 (2)	C34—C35	1.518 (4)
C14—H14A	0.9900	C34—H34A	0.9900
C14—H14B	0.9900	C34—H34B	0.9900
C15—C16	1.518 (2)	C35—C36	1.529 (6)
C15—H15A	0.9900	C35—H35A	0.9900
C15—H15B	0.9900	C35—H35B	0.9900
C16—C17	1.516 (3)	C36—H36A	0.9800
C16—H16A	0.9900	C36—H36B	0.9800
C16—H16B	0.9900	C36—H36C	0.9800
C17—C18	1.515 (3)	C32'—C33'	1.500 (13)
C17—H17A	0.9900	C32'—H32C	0.9900
C17—H17B	0.9900	C32'—H32D	0.9900
C18—H18A	0.9800	C33'—C34'	1.521 (10)
C18—H18B	0.9800	C33'—H33C	0.9900
C18—H18C	0.9800	C33'—H33D	0.9900
C19—O4	1.2641 (19)	C34'—C35'	1.504 (9)
C19—O3	1.267 (2)	C34'—H34C	0.9900
C19—C20	1.513 (2)	C34'—H34D	0.9900
C20—C21	1.530 (2)	C35'—C36'	1.500 (12)
C20—H20A	0.9900	C35'—H35C	0.9900
C20—H20B	0.9900	C35'—H35D	0.9900
C21—C22	1.526 (3)	C36'—H36D	0.9800
C21—H21A	0.9900	C36'—H36E	0.9800
C21—H21B	0.9900	C36'—H36F	0.9800
C22—C23	1.516 (3)	N1—Cu1	2.1833 (13)
C22—H22A	0.9900	N2—Cu2	2.1841 (13)
C22—H22B	0.9900	O1—Cu1	2.0006 (12)
C23—C24	1.517 (3)	O2—Cu2	1.9489 (12)
C23—H23A	0.9900	O3—Cu1	1.9497 (12)
C23—H23B	0.9900	O4—Cu2	1.9829 (12)
C24—H24A	0.9800	O5—Cu1	1.9643 (12)
C24—H24B	0.9800	O6—Cu2	1.9919 (12)
C24—H24C	0.9800	O7—Cu1	1.9781 (12)
C25—O6	1.259 (2)	O8—Cu2	1.9489 (12)
			
N1—C1—C2	122.84 (16)	C30—C29—H29A	108.9
N1—C1—H1	118.6	C28—C29—H29A	108.9
C2—C1—H1	118.6	C30—C29—H29B	108.9
C1—C2—C3	117.95 (15)	C28—C29—H29B	108.9
C1—C2—H2	121.0	H29A—C29—H29B	107.7
C3—C2—H2	121.0	C29—C30—H30A	109.5
C2—C3—C4	119.83 (15)	C29—C30—H30B	109.5
C2—C3—C6	121.12 (15)	H30A—C30—H30B	109.5
C4—C3—C6	119.05 (16)	C29—C30—H30C	109.5
C5—C4—C3	117.96 (16)	H30A—C30—H30C	109.5
C5—C4—H4	121.0	H30B—C30—H30C	109.5
C3—C4—H4	121.0	O7—C31—O8	125.79 (16)
N1—C5—C4	123.13 (15)	O7—C31—C32'	116.6 (14)
N1—C5—H5	118.4	O8—C31—C32'	117.4 (14)
C4—C5—H5	118.4	O7—C31—C32	119.2 (5)
N3—C6—C3	177.7 (2)	O8—C31—C32	114.9 (5)
N2—C7—C8	123.30 (15)	C31—C32—C33	114.1 (5)
N2—C7—H7	118.3	C31—C32—H32A	108.7
C8—C7—H7	118.3	C33—C32—H32A	108.7
C7—C8—C9	117.27 (16)	C31—C32—H32B	108.7
C7—C8—H8	121.4	C33—C32—H32B	108.7
C9—C8—H8	121.4	H32A—C32—H32B	107.6
C10—C9—C8	120.16 (14)	C32—C33—C34	113.9 (5)
C10—C9—C12	120.30 (15)	C32—C33—H33A	108.8
C8—C9—C12	119.54 (16)	C34—C33—H33A	108.8
C11—C10—C9	117.76 (15)	C32—C33—H33B	108.8
C11—C10—H10	121.1	C34—C33—H33B	108.8
C9—C10—H10	121.1	H33A—C33—H33B	107.7
N2—C11—C10	122.99 (15)	C35—C34—C33	114.0 (2)
N2—C11—H11	118.5	C35—C34—H34A	108.8
C10—C11—H11	118.5	C33—C34—H34A	108.8
N4—C12—C9	178.4 (2)	C35—C34—H34B	108.8
O1—C13—O2	125.34 (16)	C33—C34—H34B	108.8
O1—C13—C14	118.83 (15)	H34A—C34—H34B	107.7
O2—C13—C14	115.83 (14)	C34—C35—C36	113.7 (3)
C13—C14—C15	116.17 (14)	C34—C35—H35A	108.8
C13—C14—H14A	108.2	C36—C35—H35A	108.8
C15—C14—H14A	108.2	C34—C35—H35B	108.8
C13—C14—H14B	108.2	C36—C35—H35B	108.8
C15—C14—H14B	108.2	H35A—C35—H35B	107.7
H14A—C14—H14B	107.4	C35—C36—H36A	109.5
C16—C15—C14	111.59 (14)	C35—C36—H36B	109.5
C16—C15—H15A	109.3	H36A—C36—H36B	109.5
C14—C15—H15A	109.3	C35—C36—H36C	109.5
C16—C15—H15B	109.3	H36A—C36—H36C	109.5
C14—C15—H15B	109.3	H36B—C36—H36C	109.5
H15A—C15—H15B	108.0	C33'—C32'—C31	101.7 (11)
C17—C16—C15	114.64 (14)	C33'—C32'—H32C	111.4
C17—C16—H16A	108.6	C31—C32'—H32C	111.4
C15—C16—H16A	108.6	C33'—C32'—H32D	111.4
C17—C16—H16B	108.6	C31—C32'—H32D	111.4
C15—C16—H16B	108.6	H32C—C32'—H32D	109.3
H16A—C16—H16B	107.6	C32'—C33'—C34'	115.3 (15)
C18—C17—C16	112.61 (15)	C32'—C33'—H33C	108.4
C18—C17—H17A	109.1	C34'—C33'—H33C	108.4
C16—C17—H17A	109.1	C32'—C33'—H33D	108.4
C18—C17—H17B	109.1	C34'—C33'—H33D	108.4
C16—C17—H17B	109.1	H33C—C33'—H33D	107.5
H17A—C17—H17B	107.8	C35'—C34'—C33'	115.1 (7)
C17—C18—H18A	109.5	C35'—C34'—H34C	108.5
C17—C18—H18B	109.5	C33'—C34'—H34C	108.5
H18A—C18—H18B	109.5	C35'—C34'—H34D	108.5
C17—C18—H18C	109.5	C33'—C34'—H34D	108.5
H18A—C18—H18C	109.5	H34C—C34'—H34D	107.5
H18B—C18—H18C	109.5	C36'—C35'—C34'	110.4 (10)
O4—C19—O3	124.93 (15)	C36'—C35'—H35C	109.6
O4—C19—C20	118.72 (15)	C34'—C35'—H35C	109.6
O3—C19—C20	116.30 (14)	C36'—C35'—H35D	109.6
C19—C20—C21	110.45 (14)	C34'—C35'—H35D	109.6
C19—C20—H20A	109.6	H35C—C35'—H35D	108.1
C21—C20—H20A	109.6	C35'—C36'—H36D	109.5
C19—C20—H20B	109.6	C35'—C36'—H36E	109.5
C21—C20—H20B	109.6	H36D—C36'—H36E	109.5
H20A—C20—H20B	108.1	C35'—C36'—H36F	109.5
C22—C21—C20	114.11 (14)	H36D—C36'—H36F	109.5
C22—C21—H21A	108.7	H36E—C36'—H36F	109.5
C20—C21—H21A	108.7	C1—N1—C5	118.29 (14)
C22—C21—H21B	108.7	C1—N1—Cu1	124.42 (11)
C20—C21—H21B	108.7	C5—N1—Cu1	117.09 (10)
H21A—C21—H21B	107.6	C7—N2—C11	118.51 (13)
C23—C22—C21	113.86 (16)	C7—N2—Cu2	121.41 (10)
C23—C22—H22A	108.8	C11—N2—Cu2	119.80 (11)
C21—C22—H22A	108.8	C13—O1—Cu1	124.38 (11)
C23—C22—H22B	108.8	C13—O2—Cu2	120.09 (11)
C21—C22—H22B	108.8	C19—O3—Cu1	122.75 (10)
H22A—C22—H22B	107.7	C19—O4—Cu2	122.28 (11)
C22—C23—C24	113.41 (18)	C25—O5—Cu1	120.44 (11)
C22—C23—H23A	108.9	C25—O6—Cu2	124.38 (10)
C24—C23—H23A	108.9	C31—O7—Cu1	120.94 (11)
C22—C23—H23B	108.9	C31—O8—Cu2	123.24 (11)
C24—C23—H23B	108.9	O3—Cu1—O5	171.42 (5)
H23A—C23—H23B	107.7	O3—Cu1—O7	89.14 (5)
C23—C24—H24A	109.5	O5—Cu1—O7	89.11 (5)
C23—C24—H24B	109.5	O3—Cu1—O1	91.14 (5)
H24A—C24—H24B	109.5	O5—Cu1—O1	88.61 (5)
C23—C24—H24C	109.5	O7—Cu1—O1	166.54 (5)
H24A—C24—H24C	109.5	O3—Cu1—N1	97.85 (5)
H24B—C24—H24C	109.5	O5—Cu1—N1	90.72 (5)
O6—C25—O5	124.66 (15)	O7—Cu1—N1	99.76 (5)
O6—C25—C26	118.20 (14)	O1—Cu1—N1	93.53 (5)
O5—C25—C26	117.01 (14)	O3—Cu1—Cu2	84.98 (3)
C25—C26—C27	108.79 (14)	O5—Cu1—Cu2	86.49 (3)
C25—C26—H26A	109.9	O7—Cu1—Cu2	84.91 (3)
C27—C26—H26A	109.9	O1—Cu1—Cu2	81.71 (3)
C25—C26—H26B	109.9	N1—Cu1—Cu2	174.54 (4)
C27—C26—H26B	109.9	O8—Cu2—O2	171.58 (5)
H26A—C26—H26B	108.3	O8—Cu2—O4	89.69 (5)
C28—C27—C26	113.11 (14)	O2—Cu2—O4	89.73 (5)
C28—C27—H27A	109.0	O8—Cu2—O6	90.38 (5)
C26—C27—H27A	109.0	O2—Cu2—O6	88.27 (5)
C28—C27—H27B	109.0	O4—Cu2—O6	166.72 (5)
C26—C27—H27B	109.0	O8—Cu2—N2	94.24 (5)
H27A—C27—H27B	107.8	O2—Cu2—N2	94.09 (5)
C27—C28—C29	112.59 (15)	O4—Cu2—N2	101.93 (5)
C27—C28—H28A	109.1	O6—Cu2—N2	91.31 (5)
C29—C28—H28A	109.1	O8—Cu2—Cu1	84.29 (3)
C27—C28—H28B	109.1	O2—Cu2—Cu1	87.29 (3)
C29—C28—H28B	109.1	O4—Cu2—Cu1	84.23 (3)
H28A—C28—H28B	107.8	O6—Cu2—Cu1	82.56 (3)
C30—C29—C28	113.38 (16)	N2—Cu2—Cu1	173.68 (4)
			
N1—C1—C2—C3	0.5 (3)	O7—C31—C32'—C33'	−105.1 (16)
C1—C2—C3—C4	−0.6 (3)	O8—C31—C32'—C33'	80 (2)
C1—C2—C3—C6	−179.60 (16)	C32—C31—C32'—C33'	3 (10)
C2—C3—C4—C5	0.4 (3)	C31—C32'—C33'—C34'	65 (2)
C6—C3—C4—C5	179.51 (16)	C32'—C33'—C34'—C35'	176.0 (11)
C3—C4—C5—N1	−0.3 (3)	C33'—C34'—C35'—C36'	177.6 (9)
N2—C7—C8—C9	−0.4 (3)	C2—C1—N1—C5	−0.4 (3)
C7—C8—C9—C10	−0.3 (2)	C2—C1—N1—Cu1	174.28 (13)
C7—C8—C9—C12	179.51 (16)	C4—C5—N1—C1	0.2 (3)
C8—C9—C10—C11	0.8 (2)	C4—C5—N1—Cu1	−174.79 (13)
C12—C9—C10—C11	−178.94 (16)	C8—C7—N2—C11	0.5 (3)
C9—C10—C11—N2	−0.8 (3)	C8—C7—N2—Cu2	−173.47 (13)
O1—C13—C14—C15	−20.5 (2)	C10—C11—N2—C7	0.1 (2)
O2—C13—C14—C15	160.38 (17)	C10—C11—N2—Cu2	174.20 (13)
C13—C14—C15—C16	−169.24 (16)	O2—C13—O1—Cu1	−2.0 (2)
C14—C15—C16—C17	−178.66 (18)	C14—C13—O1—Cu1	178.98 (12)
C15—C16—C17—C18	−176.55 (18)	O1—C13—O2—Cu2	−8.2 (2)
O4—C19—C20—C21	111.20 (17)	C14—C13—O2—Cu2	170.82 (12)
O3—C19—C20—C21	−66.5 (2)	O4—C19—O3—Cu1	−9.1 (2)
C19—C20—C21—C22	−62.4 (2)	C20—C19—O3—Cu1	168.42 (11)
C20—C21—C22—C23	−68.0 (2)	O3—C19—O4—Cu2	1.3 (2)
C21—C22—C23—C24	176.61 (16)	C20—C19—O4—Cu2	−176.17 (11)
O6—C25—C26—C27	93.78 (18)	O6—C25—O5—Cu1	−10.5 (2)
O5—C25—C26—C27	−82.38 (18)	C26—C25—O5—Cu1	165.34 (11)
C25—C26—C27—C28	177.65 (14)	O5—C25—O6—Cu2	−0.3 (2)
C26—C27—C28—C29	−175.86 (15)	C26—C25—O6—Cu2	−176.16 (11)
C27—C28—C29—C30	170.86 (16)	O8—C31—O7—Cu1	−6.6 (2)
O7—C31—C32—C33	−118.7 (8)	C32'—C31—O7—Cu1	178.9 (8)
O8—C31—C32—C33	57.1 (10)	C32—C31—O7—Cu1	168.6 (3)
C32'—C31—C32—C33	165 (13)	O7—C31—O8—Cu2	−2.0 (2)
C31—C32—C33—C34	62.9 (10)	C32'—C31—O8—Cu2	172.4 (8)
C32—C33—C34—C35	70.1 (5)	C32—C31—O8—Cu2	−177.4 (3)
C33—C34—C35—C36	173.7 (4)		

### Hydrogen-bond geometry (Å, º)

**Table:**

*D*—H···*A*	*D*—H	H···*A*	*D*···*A*	*D*—H···*A*
C2—H2···O4^i^	0.95	2.55	3.301 (2)	137
C4—H4···N3^ii^	0.95	2.58	3.444 (2)	151
C8—H8···N4^iii^	0.95	2.48	3.422 (2)	169
C10—H10···O5^iv^	0.95	2.59	3.432 (2)	148
C20—H20*B*···O6^v^	0.99	2.66	3.479 (2)	141
C26—H26*A*···O3^vi^	0.99	2.56	3.532 (2)	167

Symmetry codes: (i) *x*, −*y*+1/2, *z*−1/2; (ii) −*x*+1, −*y*+1, −*z*+1; (iii) −*x*, −*y*, −*z*+2; (iv) *x*, −*y*+1/2, *z*+1/2; (v) *x*+1, *y*, *z*; (vi) *x*−1, *y*, *z*.
