# Supplementary figures and images for: Crystal structure of tetra­kis­(μ-caproato-κ2 O:O′)bis­[(4-cyano­pyridine-κN 1)copper(II)]
Source: Acta Crystallogr E Crystallogr Commun. 2015 Oct 14;71(Pt 11):m195–6. doi: 10.1107/S2056989015019052 (PMC4645092; doi:10.1107/S2056989015019052)

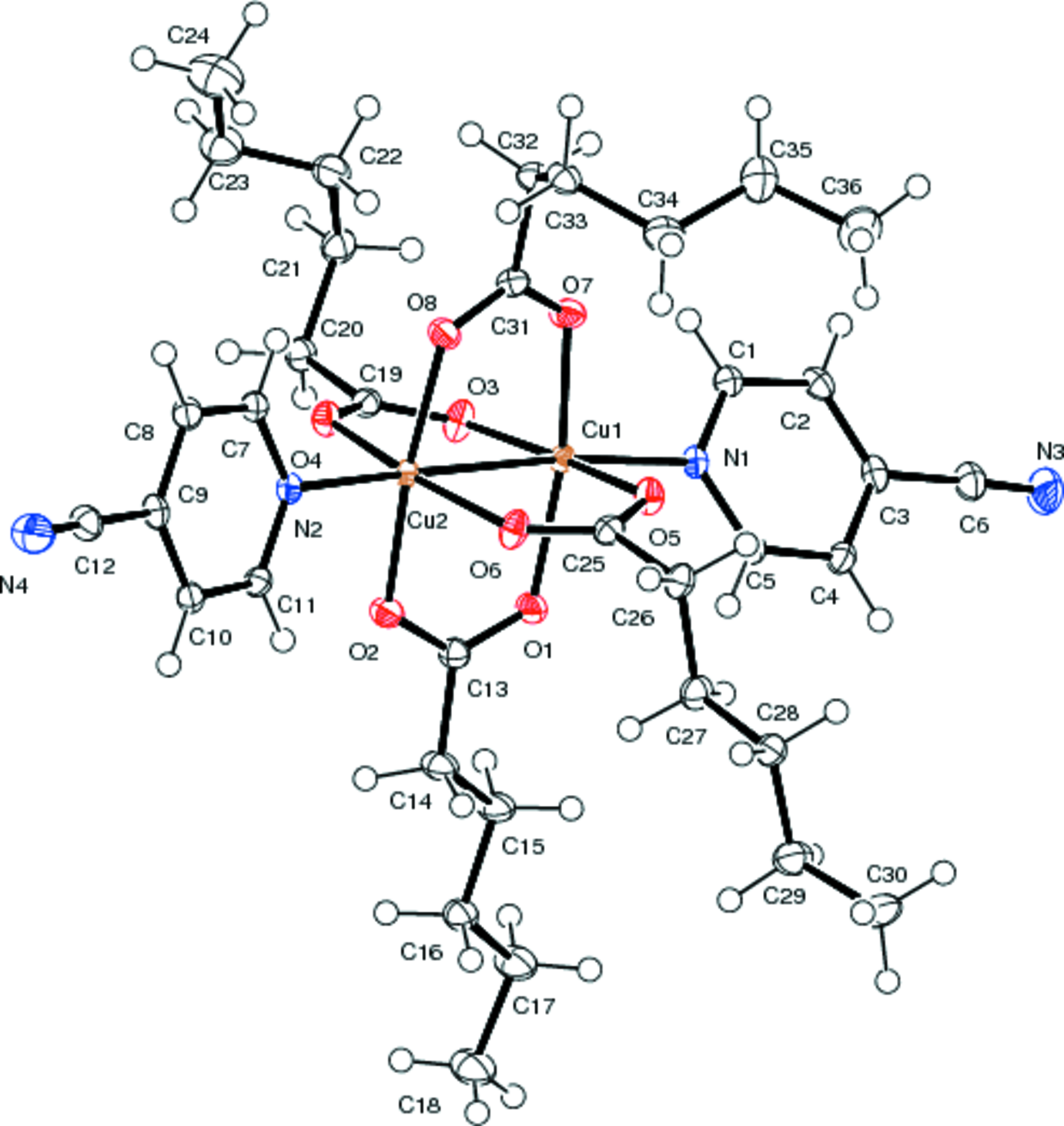

Supplement: Supplementary file 3 [file e-71-0m195-fig1.tif]

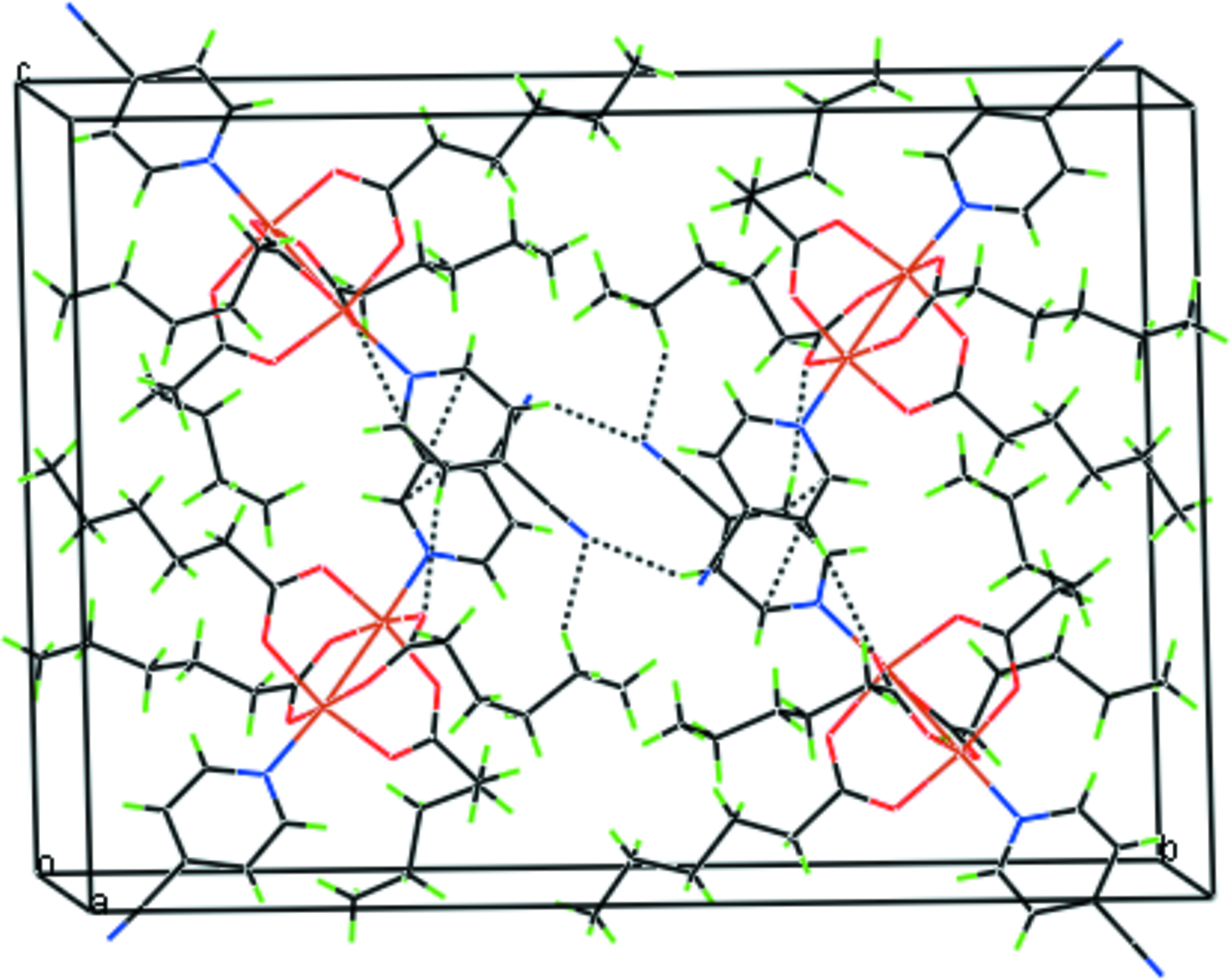

Supplement: Supplementary file 4 [file e-71-0m195-fig2.tif]
